# Recent Progress in Metal-Based Nanoparticles Mediated Photodynamic Therapy

**DOI:** 10.3390/molecules23071704

**Published:** 2018-07-12

**Authors:** Jingyao Sun, Semen Kormakov, Ying Liu, Yao Huang, Daming Wu, Zhaogang Yang

**Affiliations:** 1 College of Mechanical and Electrical Engineering, Beijing University of Chemical Technology, Beijing 100029, China; sunjingyao5566@sina.com (J.S.); s_kormakov@bk.ru (S.K.); hy06@163.com (Y.H.); 2 Department of Chemical and Biomolecular Engineering, The Ohio State University, Columbus, OH 43210, USA; 3 State Key Laboratory of Organic-Inorganic Composites, Beijing 100029, China; liuying@mail.buct.edu.cn

**Keywords:** photodynamic therapy, metal-based nanoparticles, potential toxicity

## Abstract

Photodynamic therapy (PDT) is able to non-invasively treat and diagnose various cancers and nonmalignant diseases by combining light, oxygen, and photosensitizers (PSs). However, the application of PDT is hindered by poor water solubility and limited light-penetration depth of the currently available photosensitizers (PSs). Water solubility of PSs is crucial for designing pharmaceutical formulation and administration routes. Wavelength of light source at visible range normally has therapeutic depth less than 1 mm. In this review, focus is on the recent research progress of metal-based nanoparticles being applied in PDT. The potential toxicity of these nanoscales and future directions are further discussed.

## 1. Introduction

Photodynamic therapy (PDT) is a modern and rapidly developing method for the diagnosis and treatment of a wide range of diseases from cancer treatment to root canal treatment, and with an increasing popularity owing to its antibacterial effect [1,2,3]. PDT involves the joint action of chemotherapeutic and physical (laser radiation, radiation of LEDs and other sources) factors in the presence of oxygen. The method is based on selective accumulation of photosensitizer in the tumor tissue, which is capable of generating cytotoxic agents that cause the death of tumor cells under local exposure to light with a wavelength corresponding to its maximum absorption [4,5]. The sensitizer is injected into the body, most often intravenously, and accumulates in the tissues of the tumor. Then, affected by the pathological process, the tissues are irradiated with light. The absorption of light quanta photosensitizer molecules in the presence of oxygen leads to a photochemical reaction, which results in the formation of singlet oxygen, causing tumor cell necrosis. In the early stages of tumor development, its cells are fed and oxygen by diffusion, but as the tissue grows there is a need for blood supply. The walls of the newly formed vessels are not as strong as in healthy vessels, so it is necessary to use nanoparticles that can penetrate the walls of the newly formed vessels and accumulate in the tumor tissues.

PDT is also used for the treatment of infectious agents in addition to the use of chemotherapy as one of the new effective antimicrobial techniques [6,7]. The basis of such therapy are photosensitizers, which are specific substances characterized by selective sensitivity to certain wavelengths of the optical range [8]. Photosensitizers and light irradiation taken separately do not have a therapeutic effect on the affected tissue [9]. The most significant component having an influential impact on photodynamic therapy process is photosensitizer. Appropriate choice of the substance is a guarantee of success. Photosensitizers should be characterized by i.e., selectivity for tumor cells, formation of a long-lived triplet excited state in reaction, activation with wavelength appropriate for tissue, and high chemical purity. The most important of these are activation wavelength and period of photo-sensitivity [10].

Today there are more than 1000 known photosensitizers that have both natural and synthetic origin (chlorophyll, phycobilin, porphyrins and intermediate products of their synthesis, some antibiotics, quinine, Riboflavin and several other drugs). Such photosensitizers should have the following characteristics: chemical purity and uniformity of composition; lack of dark toxicity; high ability to accumulate in the target tissue; rapid elimination from the patient’s body; high photochemical activity characterized by high quantum yield of singlet oxygen; absorption of light in the long-wave part of the spectrum (600–800 nm), in which biological tissues are most transparent, with a high coefficient of extinction [11,12,13].

The mechanism of action of PDT is that when the photosensitizer molecule absorbs a quantum of light, it goes to an excited triplet state and enters into photochemical reactions of two types. In the first type of reactions there is an interaction directly with the molecules of the biological substrate, which ultimately leads to the formation of free radicals. In the second type of reactions there takes place interaction of the excited photosensitizer with oxygen molecule to form singlet oxygen which is cytotoxic for living cells due to its property of strong oxidizer [14]. The use of PDT is not limited to oncology. This is due to the fact that most PS are able to accumulate not only in the tumor area, but also in areas with some other pathologies. These pathologies include hyperplasia, metaplasia, and inflammation. In recent years, PDT has been used to treat infectious diseases caused by bacterial and fungal infections [15]. Efficacy of PDT is also shown for the treatment and prevention of several cardiovascular diseases, and blood sterilization [16].

Currently, the technology of PDT is becoming increasingly used in modern medicine. However, there are many factors that require further study in this area. Many modern scientists are working on optimizing the mechanism of delivery of photosensitizers to target tissues, accelerating and improving the impact of active substances on the body, reducing toxicity and accelerating the withdrawal of drugs after the procedure. A separate area of research in this area is the use of metal-based nanoparticles.

**Figure 1:**
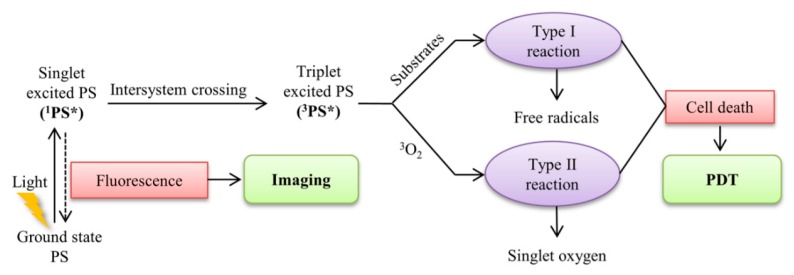
The schematic illustration of a typical photodynamic reaction (Reproduced with permission [18]).

The mechanism of photodynamic action is complex and not fully understood. It is known that singlet oxygen plays the main role in PDT, which is formed in the molecules of lipids and proteins of cell membranes and intracellular organelles when exposed to them by the quantum of light [17]. When the light absorption of the molecule PS also moving from the core to an excited state. The excited light molecules, or quantum emission of fluorescence, enter into photochemical reactions of Type I or II (as shown in Figure 1). In Type I, PS molecules interact directly with tumor tissue molecules, forming intermediate radical products that then react with oxygen, which leads to the formation of various highly active substances, primarily active forms of oxygen, entering into further redox reactions. In this case, peroxide radicals, superoxide anion, hydroxyl radical are formed, lipid peroxidation is activated, and cell membranes are damaged in violation of their functions. In a photodynamic reaction of Type I, the photosensitizer molecule is excited and passes from the ground state first to the singlet state and to the triplet excited state. In Type II, PS molecules react first with oxygen, converting it into a highly active singlet form. It interacts with proteins, nucleic acids, and lipids of cell membranes, causing their death by necrosis or apoptosis.

However, not all possible reactions explaining the mechanism of PDT have been studied and understood. There are a number of contradictory experimental data. In Reference [19] a fundamentally different approach to PDT was proposed, based on the use of endogenous mechanisms for inducing photosensitivity. The idea was to create conditions in the body in which there would be an excessive amount of synthesis of endogenous porphyrins in tumor tissues. To this end, patients were orally administered d-aminolevulinic acid, which in itself is not PS and does not accumulate in cells, but a natural precursor of protoporphyrin IX. With exogenous administration of acid, protoporphyrin IX accumulates in tumor cells. Protoporphyrin IX is a sufficiently active PS with a maximum absorption at a wavelength of 630 nm, capable of actively generating singlet oxygen. ALC-based PS has been successfully used for the diagnosis and treatment of keratosis, bladder cancer, and brain tumors. Thus, photodynamic reactions of Type I and II lead to the formation of high toxicity, the development of chain oxidation processes and, as a consequence, to the destruction of vital structures of cells and their death. These types of reactions can occur simultaneously. The advantage of this or that type of reaction is determined by the chemical structure of the photosensitizer, its concentration, the presence of extinguishers, as well as the ratio of the molecular oxygen content and the oxidized substrate in the tissues [20]. In the study of the distribution of PS in the body, it was noted that, in addition to the tissue of skin tumors, many of them are retained in high concentrations in the cells of the reticuloendothelial system, liver, kidneys, spleen, and inflamed tissues. This was a prerequisite for the study of the use of PDT in a new direction: for the treatment of diseases of non-tumor nature.

## 2. Recent Progress of Photodynamic Therapy

PDT is a method of local activation accumulated in the tissue fluorescent dye-photosensitizer by visible light in the presence of oxygen in the tissues, which leads to the development of free radical reactions and, ultimately, to the death of target cells. Assessing inequality in the distribution of such dyes in normal and pathological-modified tissue is based on fluorescent diagnostics [21,22]. Reactions underlying modern medical fields of PDT were used tens of centuries ago. The Egyptian papyri and ancient Indian medical literature describes the treatment of skin diseases, in particular vitiligo, with herbal preparations based on St. John’s wort, caraway, parsley and parsnip. It is known that these plants contain photoactive compounds, derivatives of coumarins-psoralenes [23,24]. Isolated plant preparations were used internally or locally, and the subsequent insolation of pathological areas with bright sunlight contributed to the development of photosensitizing reactions [25,26]. The development of a modern approach to the study of photosensitizers and their impact on biological objects began with Reference [27], which described the death of Paramecia in an environment with small concentrations of dyes such as acridine, eosin, fluorescin, when exposed to sunlight, while in the dark cell death was not observed.

The first clinically approved photosensitizer, known as the “hematoporphyrin derivative” or HpD, did not have a strictly defined chemical composition, but was a mixture of many porphyrins, including hematoporphyrin, protoporphyrin, deuteroporphyrin, their derivatives, monomers, dimers and oligomers and their esters. Photodynamic properties of hematoporphyrin, which became the basis for the first generation of clinical photosensitizers, were first discovered and published in 1911 in the work [28]. The first person who experienced the effect of hematoporphyrin on the human body was Meyer-Betz in 1912, when he injected himself with intravenous hematoporphyrin, resulting in swelling and pigmentation under the influence of sunlight were lasted for 2 months [29].

The ability of hematoporphyrin to selectively accumulate in the tumor was shown in Reference [30]. This work opened the possibility of using this compound for photodynamic therapy and fluorescence imaging of malignant neoplasms. Widespread PDT began in the second half of the 1970s, which is associated with the appearance of works [31,32]. These works reported the results of a successful application of a hematoporphyrin derivative in PDT for the treatment of patients with skin and basal cell carcinoma, melanoma metastases and breast cancer [33,34,35]. Currently, there is active research in the field of development of PDT. At the beginning of this century, new methods of diagnosis and treatment of tumors of the colon and bladder, tumors of the brain and spinal cord, new methods of treatment in skin and plastic surgery and cosmetology were developed [36,37,38]. To increase the efficiency of PDT, compounds were proposed with greater selectivity of accumulation in tumor tissue, better photosensitizing properties and providing an increase in the depth of photodynamic action by shifting the absorption maxima into a longer-wave region of the spectrum (more than 650 nm) compared to first-generation drugs. Due to the individual characteristics of the patient’s body, and considering the conditions of the disease, therapy requires the selection of optimal drugs for treatment. In this regard, a large amount of scientific work aims to study the most common and effective photosensitizers [39]. Ideally, this drug should meet the following requirements: to reliably generate a photodynamic reaction, to be hydrophilic for easy systemic application and nontoxic to activated light, to clinically activate beneficial light wavelengths. The drug should be well distributed on target tissues and leave the body quickly and completely after the procedure [40,41].

Another important factor in the successful use of PDT is the use of the most effective light source to activate the photosensitizers [42]. The light source must ensure the penetration of light to the required depth into the tissues, provide full and uniform illumination of the required zone, the wavelength of the light corresponding to the maximum absorption of the active substance. In this regard, the choice of light source depends not only on the choice of drugs, but also on the depth, size, and characteristics of target tissues [43,44,45]. The development of PDT technology can aid in the fight against a range of diseases, such as tumors of the colon and bladder, tumors of the brain and spinal cord, and to develop new methods of treatment of skin in plastic surgery and cosmetology [46,47]. Positive results of treatment of proinflammatory diseases by photodynamic therapy show high efficiency of this method in respect of aerobic, facultative, and obligate anaerobic bacteria, and microscopic fungi [48,49].

Photodynamic therapy includes the following mechanism of action: photosensitizer absorbs the energy of the same wavelength from the light source, transmits this energy to the substrate, and destroys the microorganisms by irreversibly oxidizing the cellular components through formation of short-lived reactive molecules [50,51]. Photodynamic action can cause different types of cell death: apoptosis, necrosis, and autophagy. For the development of apoptosis, it is necessary to preserve the integrity of the plasma membrane and a sufficient level of ATP. Chromatin condenses and forms apoptotic cells, and DNA fragmentation occurs when apoptotic cells die. This process of self-destruction of the cell is strictly controlled at the level of regulatory proteins and participating effector enzymes. Proteolytic caspases play a key role in apoptosis. Caspase activation can be initiated both outside and inside the cells. In the first case, the start of the cascade begins with the activation of one of the receptors located on the cell membrane, which perceives the external signal (for example, Fas, TNF, DR-4, DR-5). However, in the second case, which is most likely under photodynamic action, signals for starting apoptosis can come from mitochondria, electron paramagnetic resonance (EPR) and lysosomes. Necrosis is a passive process that does not require energy. In necrosis, there is a violation of the integrity and, accordingly, permeability of the membrane, protein denaturation, and output of the cellular content in the external environment. In the case of autophagy in the cytoplasm of cells, accumulation of membrane bubbles occurs that contain fragments of organelles. When you merge autophagosome with lysosomes, autophagosomes are formed which digest the contents [52,53].

The study of the mechanisms of intracellular and intercellular distribution of photosensitizers is an important step in the development of new drugs for PDT. Knowledge of these mechanisms allows for increased efficiency of photodynamic influence on pathological tissues, to predict toxic properties of PS and, thus, to minimize degree of negative influence on normal organs and tissues. Since the basic effector of photodynamic therapy singlet oxygen in its short life (less than 0.04 µs) diffuses in the cell by no more than 0.02 µm, it is capable of exerting mainly local effects of the PS molecule mucus. Singlet oxygen in cells oxidizes first of all amino acids as a part of proteins (tryptophan, histidine, methionine, cysteine, etc.), ascorbate, and sugars and nucleotides, which are much worse than lipids [54,55]. The radius of cytotoxic action of singlet oxygen to the cell does not exceed 0.01–0.02 microns, and its life expectancy in biological systems is less than 0.04 µm [56], the small radius of cytotoxic action of singlet oxygen determines the locality of the action, since it damages only biostructures that are in the vicinity of molecules of the photosensitizer. Therefore, the localization of the photosensitizer plays a crucial role in the mechanism of photo-damage, determining intracellular and tissue targets, which will primarily be exposed to photodynamic effects [57]. With the introduction of photosensitizer in the bloodstream, they bind to whey proteins—albumins, globulins, lipoproteins of low or high density—and form complexes, and only a small part of the PS can remain free [58,59]. The possibility of binding to certain whey proteins affects the polarity of the PS.

With an increase in PS hydrophobicity, there is an increase in the probability of binding the dye to low- and high-density lipoproteins [60]. The PS complexes formed with proteins are absorbed by endothelial cells in the capillaries of the bloodstream, after which there is a binding of the dyes with the adventitia of the vessels and the arrival of PS in the extracellular matrix with subsequent accumulation in the cells [59,60]. After photoexcitation and subsequent relaxation of the molecule of the photosensitizer is returned to its original state and is able to participate again in the chemical reaction. The whole cycle can be started again after the absorption of a new quantum of light energy. However, after a certain number of cycles, the photosensitizer “burns out”, i.e., loses the ability to participate in the photodynamic reaction. This effect is called photobleaching [61].

After intravenous injection the highest concentration of photosensitizer is observed in the liver, kidneys, spleen, and heart, as these organs are characterized by a high level of blood supply and the presence of perforated capillaries. There is a redistribution of PS to other organs and tissues, such as the lungs, intestines, stomach, and skin. The lowest level of accumulation of photosensitizers is noted in the muscles [62].The ways to remove the photosensitizer from the patient’s body are determined by the chemical structure of the drug. As a rule, hydrophobic PS are excreted with faeces and bile through the liver, and hydrophilic with urine [63]. Tumor tissues have an increased disposition to the accumulation of PS [64]. This may be due to a number of reasons:

(1) Tumor vessels have increased permeability compared to healthy tissues. Tissue with increased vascular permeability is a weak barrier for most broadcasters moving with blood [65,66,67].
(2) Low lymph drainage characteristic of tumor area. The decrease in the drainage function of the lymphatic system contributes to the fact that photosensitizers are slowly excreted from the tumor site, which leads to their local accumulation [68].
(3) High speed of proliferation in the tumor, in which there is a high level of expression of low-density lipoprotein receptors, binding a large number of hydrophobic molecules of photosensitizer [69].
(4) Lower pH value of the tumor than in healthy tissues. The main reason for strengthening the accumulation of photosensitizers in the acidification of the environment is to increase the lipophilicity of the drug, if protonated [70].
(5) Abnormal structure of the tumor stroma, characterized by increased intercellular space and increased production of collagen, which binds porphyrins [71].
(6) Large number of macrophages in tumor tissue, which are effective traps for hydrophobic photosensitizers [72].

Photosensitizers, received from vessels in the extracellular matrix, can penetrate into the cell either by diffusion or by receptor-mediated endocytosis (clathrin- and caveolin-dependent pathways). Large aggregates or particles containing PS can be absorbed by the cell by phagocytosis. In the case of endocytosis photosensitizer mainly enters lysosomes. The specific method of penetration of PS into the cell depends primarily on the size of molecules and their ability to aggregate. Modern scientific literature provides a detailed description of the mechanism of penetration of various sensitizers in tumor cells by diffusion [73], endocytosis [74] and phagocytosis [75]. In References [76,77] it was shown that after the PDT procedure it is possible to recalibrate the PS. This phenomenon can cause damage to non-target subcellular structures.

Currently, PDT has attracted a lot of attention as a noninvasive and safe method for the treatment of cancer [78,79]. Special attention is paid to the study of the possibility of using free radicals in the treatment of diseases. According to commonly knowledge of free radicals, these reactive forms are harmful for a whole organism. The molecules attack tissues at the cellular level leading to numerous mutations, and this leads to diseases such as neurodegenerative disorders, diabetes, cardiovascular, cancers etc. [80]. Considering the high reactivity of free radicals and their high possibility of damaging cells, these forms can be influential for anticancer therapy. Current medicine pays increasingly more attention to photodynamic therapy as a promising direction for cancer treatment. Based on the physical and photochemical basis of the aforementioned PDT treatment, the basis of treatment is the ability to attack and extinguish the free radical generation of cancers cells. PDT is considered to be a safe and promising therapy. Nevertheless, this action is limited by naturally occurring defense systems which unfortunately help the cancer cells survive and scavenge the radicals. Examples are superoxide dismutase and glutathione which uphold the normal functioning organism and counteract the negative influence of free radicals and oxidative stress [81]. Some terms should be retained for cancer treatment. Firstly, the therapy should lead to irreversible damage of the cancer cells, the destruction should not include healthy tissues/cells and, most significantly, the damage must lead to the total destruction of tumor cells present in concrete tissue [82]. In view of this, a promising approach seems to be in connection of traditional therapy (i.e., chemotherapy) with PDT. Continuous exploration in this medical field allows us to infer the effects of synergy [83]. The most significant cases of the use of PDT and its connection with other antitumor therapies are presented below.

Cancer treatment is not the only application of this technique in medicine: due to the presence of its antibacterial effect, therapy can be applied to many diseases, including root canal treatment of purulent diseases, anti-acne therapy, psoriasis and herpes therapy and physical damage [84]. The progress of scientists in the field of development of light sources and synthesis of new active substances suggests that PDT is one of the most promising and quickly developing techniques in the diagnosis and treatment of various diseases.

## 3. Applications of Metal-Based Nanoparticles in PDT

It is no secret that currently the use of nanotechnology is gaining in popularity and affects an increasing number of scientific fields [85,86,87,88,89,90,91,92,93]. Nanomaterials are usually easily able to form complex compounds with other substances, including organic materials. At the same time, the developed surface of nanoparticles leads to its increased chemical activity, which makes it possible to use oxide nanoparticles to suppress the growth of pathogenic bacteria, including those resistant to antibiotics [94,95]. Irradiation of nanoparticles with relatively low-power radiation can lead to photostimulated reactions on the surface of nanoparticles. Such reactions—in particular, the generation of singlet oxygen—are used in photodynamic therapy. Formed complexes possess new properties. Therefore, nanoparticles can be in contact with nucleic acids and proteins embedded in membranes, to penetrate into cell organelles by altering the functions of the bio-structures [96,97]. The development of knowledge in the application of nanoparticles has led to the possibility of using this technology in the PDT method [98]. Recent studies have shown that metal-based nanoparticles can be used as photosensitizers, delivery vehicles, and upconversion tools [99]. Dispersions, suspensions, and sols of metal nanoparticles are of interest among various forms of nanoscale material use. Their advantage lies in their relatively narrow size and shape distribution and long period of activity. There is reason to believe that stable nanoparticles of metals in water dispersions will find useful applications in biology and medicine. Currently, a number of studies are being carried out to research the possibility of using nanoparticles based on molybdenum oxide, TiO_2_, ZnO, and tungsten oxide as photosensitizers in PDT [100,101,102].

### 3.1. Gold Nanoparticles

Gold is one of the most popular materials for nanoparticles used in medicine [103]. One of the directions in cancer therapy is connected with the heating of gold particles by IR laser radiation. A special feature of metal nanoparticles is the presence of resonant absorption of electromagnetic energy for cases when the size of the nanoparticles is much smaller than the wavelength. This absorption is associated with the surface plasmon resonance, which is the collective oscillation of electron gas on the surface of the nanoparticle. For most metals, the wavelength of plasmon resonance lies in the region of visible and shorter waves. For example, the surface plasmon resonance of a gold nanostar placed in water lies close to 520 nm [104].

**Figure 2:**
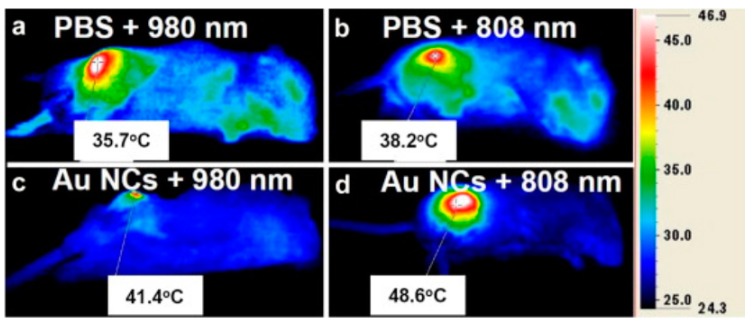
In vivo photothermal images of mice treated with phosphate buffer solution (PBS) and Au nanoparticles under 808, 915 and 980 nm laser (150 mW/cm^2^ power) (Reproduced with permission [110]).

The technology of heating gold nanoparticles by infrared laser radiation for the purpose of local thermal damage of cells was first proposed in Reference [105]. The successful application of this technology in the field of cancer treatment was considered in Reference [106]. Due to the fact that the heating of gold nanoparticles requires a wavelength of about 520 nm, these characteristics do not allow light to penetrate deep into the tissue, for which the most optimal wavelengths are 800–900 nm. This basic research aims to study the synthesis of nanoparticles at surface resonance wavelengths, corresponding to the maximum transparency of body tissue [107,108]. The light-absorbing properties of gold nanoparticles (the localized surface plasmon resonance) can be easily regulated by controlling the morphology of the size and shape of the nanostructure of the material in the synthesis process [109]. In recent works, leading scientists have proven that a change in the structure of gold nanoparticles allows to change their features to achieve the most efficient use of this material as a photosensitizer [110,111]. The results of Figure 2 shows that the temperature of mice treated with Au nanoparticles increased significantly under light irradiation. The temperature changes of melanoma tumor transplanted mice under 808 and 980 nm laser irradiation were 12.6 and 10.5 °C, respectively [110]. The results of 808 nm laser irradiation are caused by nanomaterial-mediated photothermal (NmPTT) effect, while the results of the 980 nm irradiation are considered to be the result of the combination of nanomaterial-mediated photodynamic therapeutic (NmPDT) and NmPTT effects.

In Reference [112], gold nanoparticles with a particle size of 2–4 nm were obtained using a hydrophobic thiol group containing PS, namely phthalocyanine (PC), as a stabilizing agent. It should also be noted that gold nanoparticles are not only photosensitizers, but also have found considerable use in the field of delivery vehicles. In Reference [113], it was shown that, due to targeted delivery through the use of gold nanoparticles, the efficiency of photodynamic therapy has significantly improved. In addition, it did not reveal any toxic action of the conjugates if introduced into the body at therapeutic doses. These results make it possible to conclude that gold is one of the most promising materials in the field of development of PDT technology [114].

### 3.2. Silver Nanoparticles

Silver is one of the strongest-known natural antibiotics and has been used by humans to kill a variety of microorganisms for many years. Colloidal nanosilver is a product consisting of microscopic silver nanoparticles suspended in demineralized and deionized water. Typical silver nanoparticles have a size of 20–25 nm. They have an extremely large specific surface area, which increases the contact area of silver with bacteria or viruses, significantly improving its bactericidal action. Thus, the use of silver in the form of nanoparticles allows the concentration of silver to reduce hundreds of times while maintaining all bactericidal properties. In Reference [115], a wide-ranging study was conducted to examine the effect of silver ions on bacteria. The use of nanoparticles of noble metals (including silver) has found wide application as a technology of container delivery of photosensitizers to target tissues. The action of silver nanoparticles in combination with conjugation in such nanocontainers of photosensitizers can lead to a significant increase in the efficiency of fault detection and isolation (FDI) microorganisms due to the bimodal action of such nanocomposites [116,117]. The formation and stabilization of nanosized colloidal metal particles demands careful attention to the preparation conditions and to the presence of stabilizers. Nanoparticles of silver, gold, platinum, and copper have been prepared by various methods, but most of their shapes have been limited to spheres [118]. Thus, silver nanoparticles are a promising and effective approach to enhance photodynamic action on microorganisms, as well as their safety for mammalian cells for photodynamic therapy of tumors.

### 3.3. Copper-Based Nanoparticles

**Figure 3:**
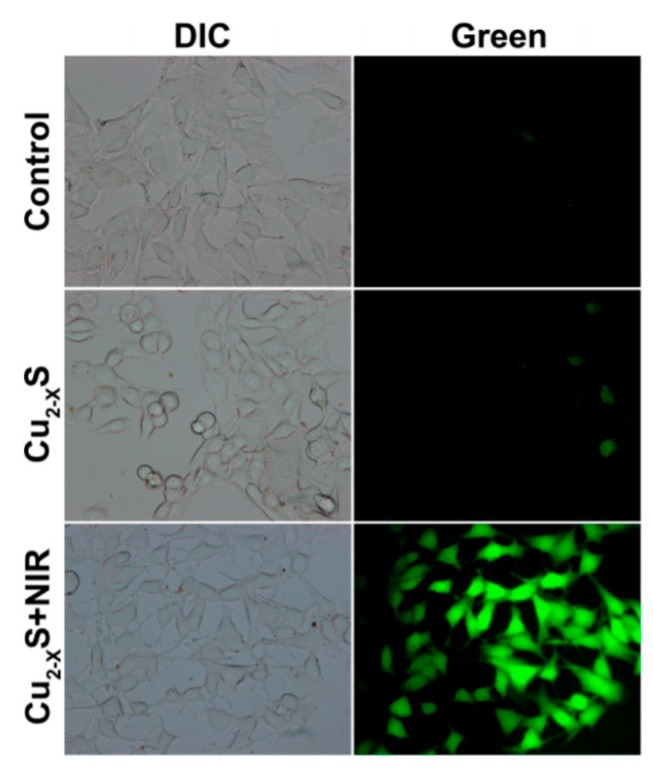
Visible light and fluorescence images of B16 cells incubated with plasmonic Cu_2−x_S nanocrystals for 6 h (Reproduced with permission [120]).

Copper is a trace element vital for the human body. According to recommendations, the daily demand for copper is about 900 µg. When copper deficiency decreases phagocytic activity of granulocytes and synthesis of immunoglobulins, immunodeficiency occurs. An important biological role of copper is to participate in the processes of proliferation and differentiation of cells. It is experimentally proved that copper gluconate (Cu^2+^) in immunodeficiency contribute to the increase of IgG level, prevent the appearance of malignant cells, and enhance the effect of anticancer protection. On the contrary, copper deficiency increases the probability of neoplasms. During the course of pathological processes, the body accumulates information for the bank of immunological memory. As a result, specific antibodies in the synthesis involve copper. In case of repeated penetration into the body of a known antigen, the acquired immunity is used, so the immune reaction proceeds faster and more clearly, i.e., copper has immunomodulatory properties [119]. Copper sulfide nanoparticles are also widely used in the field of PDT techniques. In modern research both the drug-delivery property in the target tissue and the photodynamic activity of the copper-based particles themselves are described in detail in Reference [120]. In Figure 3, B16 cells treated with plasmonic copper sulfide (Cu_2−x_S) showed stronger 2,7-dichlorofluorescein (DCF) fluorescence signal under near infrared light irradiation. The enhancement of the signal intensity is in accordance with the results of electron spin resonance and fluorescence analysis. It is proved that Cu_2−x_S nanocrystal is involved in the PDT process and can only induce the reactive oxygen species to trigger the biological reaction under the condition of near-infrared light irradiation.

CuS nanoparticles have a broad absorption from 700 to 1100 nm. This property allows heat to be generated by particles and near-infrared light, which can be harnessed to kill cancer cells [121]. In Reference [122] it was shown that CuS nanoparticles are effective agents for both PTT and PDT. The CuS nanoparticles produce both heat and reactive oxygen species when excited by laser and show strong anticancer effects. In Reference [123] it was proposed to use a new method of synthesis of gold nanocubes based on copper oxide nanoparticles. This method differs in economic efficiency and allows the use of the received particles as agents for carrying out photothermal therapy with simultaneous visualization of process by a photoacoustic method.

As a new type of agent for treatment of cancer, CuS nanoparticles are characterized by their low cost, simple and easy preparation as well as small size for surface modification. The other efficient cancer treatment method is PDT. The photosensitizers (PSs) are used to generate highly reactive oxygen species (ROS) including hydroxyl radicals (%OH), singlet oxygen (1O_2_), and peroxides (R-O-O%) for destroying cancer cells by photoexcitation [124,125]. For clinical applications, ideal PSs are supposed to occur at a wavelength between 700 and 1000 nm since human tissue is penetrable at this energy level. CuS nanoparticles are gaining more and more attention in PTT and PDT for their unique physicochemical characteristic [126]. However, the mechanisms for CuS nanoparticles as PTT and PDT agents are not yet clear. In this paper, we will concentrate on the mechanism studies by evaluation CuS nanoparticles as effective agents for simultaneous PTT and PDT on cancers.

### 3.4. Magnetic Nanoparticles

Magnetic nanoparticles are of particular interest for research and have great potential in their application in biology and medicine. These nanoparticles can affect certain target tissues in the body, without having a toxic effect on healthy tissues. In order to control and contain these particles, an adjustable magnetic field orientation is applied [127]. Toxicity of oxide nanoparticles is low in comparison with metal nanoparticles, therefore they are used for realization of unique methods, such as delivery vehicles in thermal therapy, when heated by laser or microwave radiation to the destruction temperature of the pathological tissue, etc. [128,129,130]. Various particles coated with a metal shell are usually used under the action of magnetic nanoparticles. The greatest attention is on particles with a shell of magnetite Fe_3_O_4_, which, thanks to their spherical shape and narrow size distribution, have found wide application in the diagnosis and treatment of various diseases [131]. In Reference [131], a study of the effects of chemo- and photothermal therapy of cancer tumors was conducted. Mesoporous magnetic nanoclusters of gold were used as carriers and therapeutic agents. As a result of the study, mice were found to have significant inhibition of tumor growth and metastasis due to the therapeutic effect and targeted delivery of gold nanoclusters.

### 3.5. Metal-Organic Frameworks in PDT

Metal-organic frameworks (MOFs) are organic-inorganic hybrid materials, which form through the self-assembly of organic ligands and metallic clusters through the coordination bonds with intramolecular pores. The arrangement of organic ligands and metallic clusters has obvious directivity, which can form different adsorption properties, optical properties, and electromagnetic properties. The MOFs have the advantages of high porosity, low density, large specific surface area, regular channel, adjustable aperture, and diverse topology and tailoring.

In recent years, MOFs have been used in PDT. Nanoscale metal-organic frameworks (NMOFs) have shown great potential in biomedicine owing to their structural/chemical diversities, high molecular loading capacities, and intrinsic biodegradability. In 2014, Lu et al. [132] reported the first application of nanoscaled MOF in PDT. They reported the rational design of a Hf–porphyrin nanoscale metal–organic framework, DBP–UiO, as an exceptionally effective photosensitizer for PDT of resistant head and neck cancer. DBP–UiO displayed greatly enhanced PDT efficacy both in vitro and in vivo, leading to complete tumor eradication in half of the mice receiving a single DBP–UiO dose and a single light exposure. After that, more applications of nanoscaled MOFs in PDT were reported [36,133]. The MOFs have already become a new type of representative metal-based nanoparticles applied in PDT applications.

## 4. Potential Toxicity of Metal-Based Nanoparticles

Metal nanoparticles have received increasing interest in many fields, including the PDT applications mainly discussed in this review. However, due to their special physical and chemical properties, nanoparticles may have adverse effects on the level of subcellular and protein in organs and tissues. Properties such as chemical composition, small size, high surface-area-to-volume-ratio, aggregation behavior, functional groups and so on would significantly influence the toxicity of nanoparticles for living organisms [134,135]. When particle size decreases, some metal-based nanoparticles exhibit increased toxicity, even though the same material is relatively inert in its bulk form, such as Ag, Au and Cu. Nanoparticles also will interact with enzymes and proteins in mammalian cells, which can interfere with antioxidant defense mechanisms, lead to the generation of reactive oxygen species, the initiation of inflammatory reactions, and disturbance and destruction of mitochondria, leading to cell apoptosis or necrosis. Thus, there is still a lot of work needed to be done to recognize the potential toxicity of metal-based nanoparticles in PDT applications and determine whether the benefits exceed the risks associated with them.

Compared with the photosensitizers traditionally used in photodynamic therapy, metal-based nanoparticles have the advantages of high loading, slow degradation, long cycle time, and targeted and controllable release. Thus, they are often used as target drug delivery carriers in PDT applications. However, the interaction between metal-based nanoparticles and cells needs to be considered from the entry routes of various possible pathways (e.g., through skin [136], gastrointestinal tract [137], blood circulation, lungs [138], etc.) into potential target organs. After nanoparticles enter the body circulation, they may affect the toxicity of the endothelial cell membrane and/or destroy the tight junctions of the blood-brain barrier, and then enter the brain environment [139]. Compared with larger size materials, Ag and Cu nanoparticles are more likely to enter human organs and circulatory systems, and cannot be detected by normal phagocytic defense mechanisms, causing them to enter the blood or move through the blood-brain barrier into the nervous system [140]. In addition, Ag, Cu and Al nanoparticles can induce oxidative stress reaction and produce damaging free radicals on endothelial cell membrane [139]. This interference may also lead to dysfunction of blood-brain barrier, leading to the entry of metal-based nanoparticles into the central nervous system.

In addition to penetrating the blood-brain barrier [141], metal-based nanoparticles also can penetrate the blood-testis barrier (especially Leydig cells) [142], thus causing reproductive problems. Recent studies show that certain metal-based nanoparticles can attenuated the proliferation of spermatogonial stem cells in vitro [143]. However, the interaction between metal-based nanoparticles and the normal function of human body has not been systematically studied and understood. In view of the profound impact of nanoparticles on human health, it is foreseeable that researchers will continue to systematically study and evaluate them through a variety of scientific methods. In this section, the potential toxicity of several kinds of specific metal-based nanoparticles are discussed and organized according to elemental compositions with an emphasis on the evaluation of toxicity.

### 4.1. Gold Nanoparticles

There are an increasing number of studies on the potential toxicity of Au-based nanoparticles. According to currently published data, the toxicity of Au nanoparticle is highly dependent on its synthetic methods and its shape, size, surface chemical properties and surface charge.

**Figure 4:**
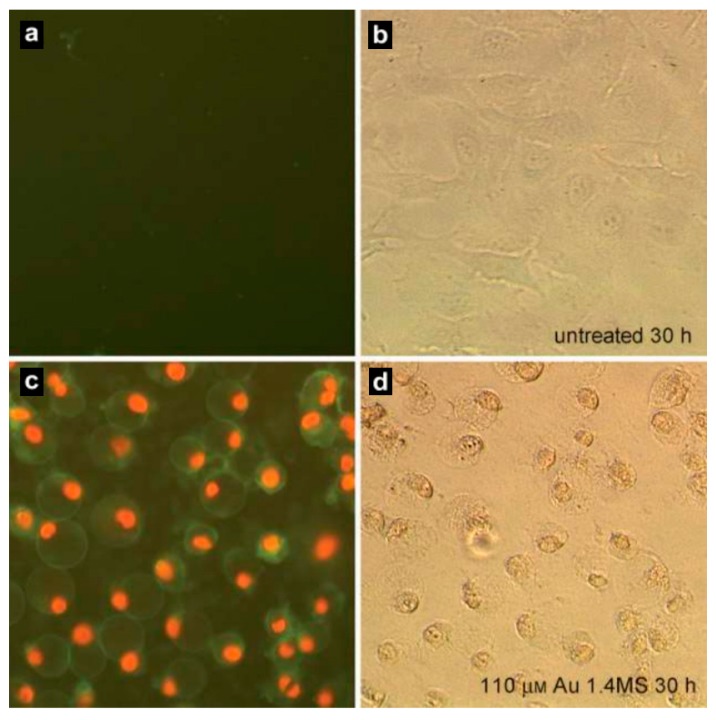
Fluorescence pictures of HeLa cells for the detection of apoptosis (annexin V, green fluorescence) and necrosis (propidium iodide, red fluorescence). (**a**,**b**) Untreated cells strained double negative for annexin V and propidium iodide, (**c**,**d**) necrotic cells presented green and red fluorescence for annexin V and propidium iodide, respectively. (Reproduced with permission [144]).

In vivo toxicity studies of intravenous colloid Au nanoparticles in mice showed that smaller particles (10~50 nm) were more toxic than larger particles (100~200 nm). Pan et al. [144] systematically studied the water-soluble gold nanoparticle of triphenylphosphine derivatives ranging in size from 0.8 to 15 nm, and tested the toxicity of these particles in four cell lines (including connective tissue fibroblasts, epithelial cells, macrophages and melanoma cells). These cell lines are most sensitive to gold nanoparticles 1.4 nm in size, which leads to a change in the IC50 value within the cell line in the range of 30 to 56 μM, depending on the combination of particular 1.4 nm gold nanoparticles and the cell line. When the size of gold nanoparticles is 15 nm, even the concentration of Tauredon (gold thiomalate) under 60 or 100 times concentration is still nontoxic. Figure 4 shows typical pictures of healthy cells and necrotic cells treated with 110 μM Au nanoparticles (1.4 nm in diameter) [144]. The untreated cells treated show double negative staining under annexin V and propidium iodide (Figure 4a,b), whereas the cells treated with Au nanoparticles present double positive (Figure 4c,d).

Wang et al. [145] studied the effect of shape of Au nanoparticles on toxicity. It was found that cetyltrimethylammonium bromide (CTAB) coated Au nanoparticles were more toxic to human HaCaT keratinocytes than spherical ones (~30 nm). MTT test, absorption spectroscopy and transmission electron microscope (TEM) were applied to analysis the cytotoxicity of gold nanomaterial. The researchers found the toxicity of Au nanoparticles is a result of a combination of factors, which is difficult to understand by single-factor analysis. The recent results from Li’s group showed that Au nanoparticles with the size of 20 nm average were nontoxic to lung fibroblasts when citric acid decreased [146]. However, the Au nanoparticles did produce a large amount of oxidative DNA damage and downregulated the DNA damage and the expression of cell-cycle genes.

There is still much controversy about whether Au nanoparticles are toxic in the academic community. In other words, although the results for toxicity of Au nanoparticles seem to be bleak, many other studies have reported them as nonreactive and nontoxic to body cells. Shukla’s group [147] reported that the toxicity of 3.5 nm Au nanoparticles lysine to macrophages was 100 μM after 72 h exposure, and there has no significant effect on the secretion of proinflammatory cytokines TNF-α or IL-1β. Furthermore, Conner and co-workers [148] also reported that spherical Au nanoparticles with different sizes (4, 12, and 18 nm) and surface modifiers are all nontoxic to human leukemic cells. These studies have tried to indicate that the surface chemistry and synthesis conditions of Au nanoparticles play important roles in modifying biological reactions.

### 4.2. Silver Nanoparticles

**Figure 5:**
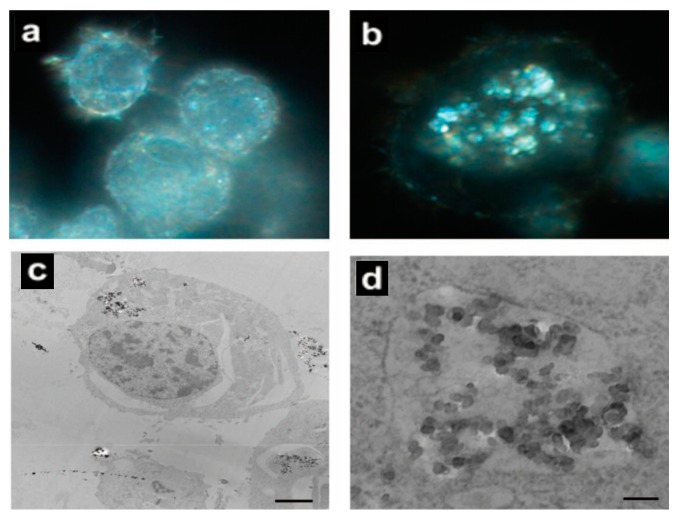
Comparative study on the uptake of Ag nanoparticles by alveolar macrophages. (**a**,**b**) Light microscope images illustrating uptake of nanoparticles in alveolar macrophages at 100× oil magnification after 6 h. (**a**) Control; (**b**) Cells treated with 30 nm Ag nanoparticles (25 µg/mL) for 6 h; (**c**,**d**) Low-magnification and high-magnification TEM images of cells that internalized 55 nm Ag nanoparticles (25 µg/mL) into vacuoles after 24 h incubation. The scale bars are 2 μm and 500 nm, respectively. (Reproduced with permission [149]).

Carlson et al. [149] found that the toxicity of silver nanoparticles is also related to size. They further found that the toxicity mechanism of silver nanoparticles is mainly mediated by oxidative stress. They systematically evaluated the effects of three known sizes of silver nanoparticles (15 nm, 30 nm and 55 nm) on cell viability. Alveolar macrophages were selected as research objects and their potential roles in the initiation of oxidative stress were studied. The cells exposed to silver nanoparticles will produce abnormal morphology and adhesion characteristics, and 24 h later, there will be obvious nanoparticle uptake. The researchers used mitochondria and cell membrane activity as well as reactive oxygen species (ROS) as toxicity assessment indexes. After 24 h of exposure, the activity index decreased sharply with the dosage increase of Ag 15 nm and Ag 30 nm nanoparticle (10–75 g/mL). When the concentration of Ag 15 nm was 50 g/mL, the level of ROS increased by more than 10 times, indicating that the cytotoxicity of Ag 15 nm might be mediated by oxidative stress. In addition, by measuring the release of cytokine/chemokine levels (including tumor necrosis factor (TNF-α), macrophage inhibitory protein (MIP-2), and interleukin-6 (IL-6)) in the medium, the activation of the release of traditional inflammatory mediators was detected. After exposure to Ag 15 nm nanoparticles for 24 h, the release of TNF-α, MIP-2, and IL-1β could show obvious inflammatory reaction. Figure 5 presents the endocytosis of Ag nanoparticles with different sizes by alveolar macrophages. Obvious uptake of Ag nanoparticles can be found after 24 h incubation, and the nanoparticles also tend to form micron-sized aggregations both outside and inside the macrophages.

Kim and colleagues [150] used Sprague–Dawley rats as research objects and tested the oral toxicity of silver nanoparticles (60 nm) for 28 days. Male and female rats were selected to conduct a controlled trial of low-dose group (30 mg/kg), medium-dose group (300 mg/kg) and high-dose group (1000 mg/kg). After 28 days of oral toxicity test, blood biochemical and hematological examinations were carried out. The histopathological examination and the distribution of silver nanoparticles in the rats’ bodies were also studied. The results showed that there was no significant variation in weight and dose of silver nanoparticles in male and female rats. However, the values of alkaline phosphatase and cholesterol in the subjects showed a significant dose-dependent variation, and silver nanoparticles more than 300 mg may lead to slight liver damage. That is, silver nanoparticles do not cause genotoxicity in male and female rats. However, the tissue distribution of silver nanoparticles did show a dose-dependent accumulation of silver in all examined tissues. At the same time, the researchers noticed a sex-related difference in the accumulation of silver nanoparticles, and the dose of silver nanoparticles in the kidney of female rats was two-times higher than that in the kidney of male rats. Similar study using Sprague–Dawley rats was also performed by Ji et al. [151].

The influence of silver nanoparticles on gene expression has also been investigated by researchers. The effects of silver nanoparticles with the diameter of 25 nm on gene expression in different brain regions of mice were systematically studied by Ali’s group [152]. Male adult C57BL/6N mice were injected with 100 mg/kg, 500 mg/kg or 1000 mg/kg doses of silver nanoparticles, respectively, and sacrificed 24 h after injection. Then the brain tissues from different regions were quickly removed and divided into caudate nucleus, frontal cortex, and hippocampus. The total RNA was isolated from the three brain regions collected, and real-time RT-PCR analysis was carried out using mouse oxidative stress and antioxidant defense arrays. The results showed that the gene expression in caudate nucleus, frontal cortex and hippocampus of mice injected with 25 nm silver nanoparticles was significantly different, which proved that the Ag nanoparticles can induce apoptosis and produce neurotoxicity by inducing altering gene expression and free radical induced oxidative stress.

### 4.3. Copper Nanoparticles

Histological analysis showed that Cu nanoparticles had serious toxicological effects and could seriously damage the kidney, liver and spleen of mice [153]. The cytotoxicity of copper nanoparticles was systematically studied by Song et al. [154]. In order to test the cytotoxicity of copper nanoparticles, the researchers added four different sizes of copper nanoparticles (25, 50, 78 and 100 nm) and a micron grade of copper particles (~500 nm) to the fish cell lines (PLHC-1 and RTH-149) and mammalian cell lines (H4IIE and HepG2), respectively. The results show that the size, morphology, and ion release of copper nanoparticles will have an important effect on their toxicity.

Some researchers have also thought it important to distinguish the effects of dissolved metal and metal-based nanoparticles. In order to solve this problem, Barber’s group [155] used zebrafish to compare the toxicity of soluble copper and copper nanoparticles (80 nm). The results showed that nanocopper was highly toxic to zebrafish. Although the copper concentration in the copper nanoparticles suspension will accumulate above a certain concentration, it is not enough to explain the mortality of zebrafish under the environment of nanocopper. Histological and biochemical analysis showed that gill was the primary aggregation organ of nanoparticles. In order to further study the effect of copper nanoparticles on the gills of zebrafish, the zebrafishes were placed in the suspension of copper nanoparticle (100 g/L concentration) and soluble copper solution at the same concentration, respectively. Under these experimental conditions, the nanoscale copper produced completely different morphological effects and global gene expression patterns in the gills, proving that the insoluble copper nanoparticles also affect the morphology of the gills of zebrafish.

**Figure 6:**
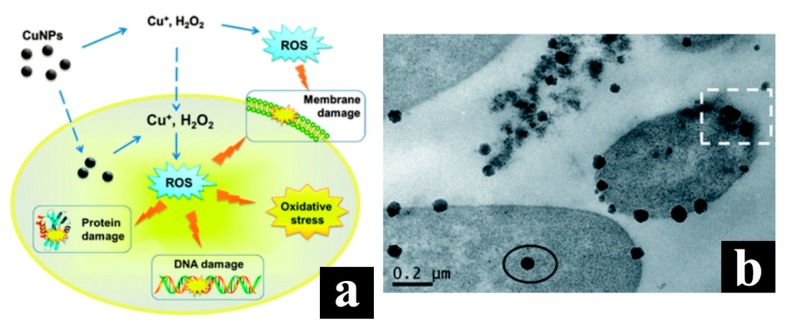
(**a**) Schematic diagram of the main toxicity mechanism of Cu nanoparticles; (**b**) Transmission electron microscope results of *Escherichia coli* cells after exposure to 80 mg·L^−1^ Cu nanoparticles for 30 min (Reproduced with permission [156]).

The sources and toxicological effects of copper nanoparticles were studied by Yao’s group [156] using stress-responsive bacterial biosensor arrays. According to the reaction of the biosensor, the researchers can induce DNA damage, protein damage and cell membrane damage in addition to inducing the oxidative stress reaction in *Escherichia coli*, and eventually cause to the inhibition of cell growth. Further studies using enzyme detoxification analysis revealed that the toxicity of copper nanoparticles was related to the formation of H_2_O_2_ (as shown in Figure 6a). In addition, the results of transmission electron microscope show that the copper nanoparticles will be adsorbed by cells and quickly phagocytic, while the copper particles in micron size are relatively stable in the cell system and will not produce toxicity, which proves the importance of toxicity assessment of materials on nanoscale. Figure 6b shows the transmission electron microscope results of *Escherichia coli* cells after exposure to 80 mg·L^−1^ Cu nanoparticles for 30 min, in which clear damage to the cell membrane can be found.

In Table 1 we list several more examples of the toxicity studies on Au, Ag, Cu, and other metal-based nanoparticles both in vitro or in vivo

## 5. Summary

PDT is undoubtedly a very promising direction for cancer treatment. However, the application of PDT is hindered by classic PSs due to their poor water solubility and limited light-penetration depth. Thus, PDT is still not recognized as a first-line treatment method. The application of nanoparticles, especially metal-based nanoparticles, in PDT is a very promising approach for the breakthrough of classic techniques in the near future. Metal-based nanoparticles can be applied as carriers of hydrophobic PSs and deliver them to the sites of different tumors via EPR effect.

In this review, we have comprehensively introduced the applications of Au, Ag, Cu and other metal-based nanoparticles in PDT. Classic PSs have defects such as low solubility, poor tumor selectivity and undesirable pharmacokinetics, but a suitable carrier platform can solve these problems to a certain extent. However, the application of metal-based nanoparticles is a double-edged sword. Researchers found they may show toxicity to cells, animals, and even humans. Although some researchers claim that the application of metal-based nanoparticles can be nontoxic under specific controlled conditions, concerns still exist and prevent them from being used in practical clinical treatments. In fact, rare metal-based nanoparticles have been approved by the FDA for clinical application. There is still a long way to go from research results to practical applications. Furthermore, we should realize that there is still great potential for efficient promotion of PDT, which can be achieved by optimizing PDT parameters, improving stability of the PS carrier, improving upconversion efficiency, etc. Since PDT involves multidisciplinary fields, frequent communication between researchers, clinicians, engineers, and industrial producers is badly needed to share and discuss cutting-edge opportunities and challenges in the field. Only in this way can PDT eventually become a frontier method for cancer diagnosis and interventional treatments with practical application values.

## Figures and Tables

**Table 1:** Selected comparative toxicity studies.

Nanoparticles	Target	Dose	Result	References
Au	Mice (in vivo)	2 × 10^5^ PPB	Uptake of nanoparticles occurred in the small intestine	[157]
Ag	Zebrafish (in vivo)	5–100 μg/mL	Dose-dependent toxicity in embryos	[158]
Ag, Cu, Al	Mice and Rat (in vivo)	30–50 mg/kg	Blood-brain barrier penetration	[159]
Ag, Mn	PC-12 cells (in vitro)	1–100 μg/mL	Cell shrinkage and irregular membrane	[160]
Ag, TiO_2_	Murine macrophage cell line (in vitro)	5 μg/mL	Aggregates of nanoparticles	[161]
TiO_2_	Mice (in vivo)	5 g/kg	Show histopathological effects	[162]
Cu, Mn	PC-12 cells (in vitro)	10 μg/mL	Genes expression altered	[163]
Al, Al_2_O_3_	Rat (in vivo)	25–250 μg/mL	Phagocytosis hindered	[164]
Cu	Zebrafish (in vivo)	0.25–1.5 mg/L	Biochemical, histopathological changes	[155]
